# Analysis of Bone Mineral Density and Bone Quality of Cortical Bone in the Human Hyoid Body and Histological Observation of the Entheses

**DOI:** 10.3390/jfb15030056

**Published:** 2024-02-22

**Authors:** Masaaki Kasahara, Tomoko Someya, Kei Kitamura, Genji Watanabe, Satoru Matsunaga, Shinichi Abe, Masayuki Hattori

**Affiliations:** 1 Department of Dental Materials Science, Tokyo Dental College, Tokyo 101-006, Japan; someyatomoko@tdc.ac.jp (T.S.); hattori@tdc.ac.jp (M.H.); 2 Department of Histology and Developmental Biology, Tokyo Dental College, Tokyo 101-006, Japan; kitamurakei@tdc.ac.jp; 3 Department of Anatomy, Tokyo Dental College, Tokyo 101-006, Japan; watanabegenji@tdc.ac.jp (G.W.); matsuna@tdc.ac.jp (S.M.); abesh@tdc.ac.jp (S.A.)

**Keywords:** hyoid bone, entheses, bone quality, apatite crystallite orientation, bone mineral density

## Abstract

The hyoid is the only bone in the human body that is completely independent, not forming a joint with any other bone; its position is maintained by the suprahyoid and infrahyoid muscles, as well as several ligaments. The purpose of this study was to ascertain the effect of the functional pressure arising from these muscles and ligaments on the hyoid body structure from its bone mineral density, bone quality, and histological observations. The area between the mesial-most part of each lesser horn and the center of the hyoid body was divided equally into four measurement regions. We conducted histological investigations at each measurement region and observed the entheses. To analyze bone mass and bone quality, we also measured bone mineral density (BMD) and analyzed biological apatite (BAp) crystallite orientation in the same regions. Histological observations identified periosteal insertions and fibrocartilaginous entheses. There was no significant difference in BMD between any of the measurement regions, but the preferential orientation of BAp crystallites was stronger in the infrahyoid muscles and ligaments, where fibrocartilaginous entheses are found, than in other places. This suggests that the functional pressure at these sites might exert a major effect not only on the morphological characteristics of the entheses but also on bone quality.

## 1. Introduction

The human hyoid bone is a horseshoe-shaped bone that is involved in major functional movements in the maxillofacial region, including respiration, mastication, and swallowing. It is also the only bone in the human body that is known to be completely independent, not forming a joint with any other bone. Ten different muscles (making a total of 20 including those on both sides) and ligaments contribute to maintaining its position [1]. The hyoid is, thus, a unique bone that is connected via these muscles and ligaments to the mandible and temporal bones, scapulae, tongue, manubrium of the sternum, and thyroid cartilage. The muscles that attach to the hyoid are broadly divided into the suprahyoid and infrahyoid muscles, the extrinsic muscles of the tongue, and the pharyngeal constrictors. Of these, the muscles with attachments to the body of the hyoid are the stylohyoid muscle, mylohyoid muscle (MH), and geniohyoid muscle (GH) among the suprahyoid muscles, and the sternohyoid muscle (SH), thyrohyoid muscle (TH) and omohyoid muscle among the infrahyoid muscles. Pearson et al. reported that the GH and MH muscles are involved in the movement of the hyoid during swallowing [2]. Gehrking et al. reported that the infrahyoid muscles depress the hyoid during swallowing and vocalization [3]. The functional pressure of the muscles from these physiological movements can be expected to have an effect on the morphology and structure of the hyoid. Capasso et al. reported that the morphology of the homo erectus hyoid differs from that of contemporary humans in having tiny greater horns and being without impressions from the attachment of the major supra-hyoid muscles [4]. They suggested that this hyoid shape is similar to those of non-human and pre-human genera and that they would have had a reduced capacity for elevating the hyoid bone compared with that of contemporary humans. Shimizu et al. suggested that the force exerted by the muscles that attach to the hyoid might affect the symphysis between the hyoid body (HB) and the greater horns in older individuals [5]. These reports indicate that the hyoid bone, which is constantly subjected to functional pressure from the suprahyoid and infrahyoid muscles, has optimized its structure and morphology to acquire the bone strength needed to cope with such a mechanical environment. However, the different types of muscle functional pressure and where and how they exert their effects remain unknown.

Recently, attention has been focused on bone quality, in the evaluation of bone. Bone quality is defined as a factor other than bone mineral density that affects bone strength as a promising index for ascertaining the relationship between the strength of bone and its structural characteristics [6]. Bone quality includes structural and material properties, with structural properties primarily including macroscopic bone structure and microscopic cortical bone porosity, and material properties including microdamage, degree of calcification, bone matrix, and bone metabolic turnover. Biological apatite (BAp) crystallite/collagen orientation in the bone matrix of material properties responds particularly strongly to local stress, and evaluating this orientation can reportedly enable the identification of regions under mechanical stress and its direction [7,8,9,10,11]. Nakano et al. used microbeam X-ray diffraction system (XRD) analysis to conduct a quantitative evaluation of BAp crystallite orientation in the trunk and limb bones of laboratory animals and found that mechanical load on the bone was strongly associated with BAp crystallite orientation [8]. A similar tendency has also been reported in human bones [9,10]. In a previous study, the authors showed that the structural characteristics of the tendon–bone entheses of the masticatory muscles in the mandibular coronoid process are associated with BAp crystallite orientation and mechanical strength and suggested that the structure and morphology of the coronoid process might be optimized to provide bone strength sufficient to withstand muscle functional pressure [12]. It should, therefore, be possible to predict the effects of functional pressure from muscles and ligaments on the local structure of the hyoid bone with a high degree of accuracy by conducting a qualitative analysis of the hyoid. Our purpose in this study was to conduct histological investigations of the entheses at the attachment sites of muscles, tendons, and ligaments to the HB, and to carry out bone mineral density and bone quality evaluations of the cortical bone at these attachment sites in order to identify the structural characteristics of the HB under the load environment imposed by the functional pressure of muscles.

## 2. Materials and Methods

### 2.1. Samples

Prior to these experiments, the hyoid bones of eight adult Japanese cadavers (mean age 82.4 ± 12.1 years; 5 men and 3 women) in the collection of the Department of Anatomy of Tokyo Dental College were harvested as specimens. To prevent spoilage, all cadavers used in the study were perfusion-fixed in 10% neutral formalin from the femoral artery and dehydrated in 70% ethanol. Only hyoid bones from patients with no history of metabolic bone disease or morphological abnormalities were used. In addition, in the women’s cadavers, those with no history of postmenopausal osteoporosis or other noted bone diseases were used. This study was approved by the Ethics Committee of Tokyo Dental College (No. 1001).

### 2.2. Preparation of Samples

**Figure 1:**
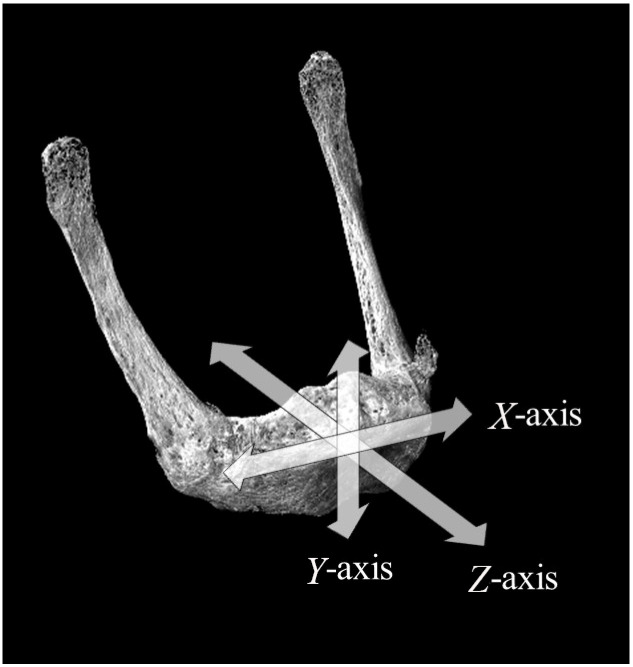
Setting of the coordinate axes.

The hyoid bones were divided into specimens for histological observation and specimens for bone mineral density (BMD) and bone quality evaluation. Before each of these evaluations, three reference axes were designated, with the *X*-axis defined as the long axis of the HB, the *Y*-axis as the direction perpendicular to the long axis of the HB, and the *Z*-axis as the anterior–posterior direction (Figure 1). The hyoid bones for use in histological observations were immersion-fixed in 4% paraformaldehyde phosphate buffer solution and decalcified in 10% ethylenediaminetetraacetic acid (EDTA) for 4 weeks. The decalcified specimens were embedded in paraffin by the usual method. Thin slices of the embedded specimens with a thickness of 5 µm were prepared as sagittal sections parallel to the *Y*-axis. Hematoxylin-eosin (H-E) staining was conducted to enable morphological histological observations, and toluidine blue staining was performed to identify the fibrocartilaginous layer (*n* = 2). All the hyoid bones for use in bone quality evaluation were scanned by microscopic computed tomography (micro-CT) (μCT-50, Scanco Medical AG, Wangen-Brüttisellen, Switzerland), their internal structure was observed, and their bone mineral density (BMD) was measured. They were then embedded in autopolymerizing resin (SCANDIPLEX, SCANDIA, Hagen, Germany), after which they were sliced with a saw microtome (SP1600, Leica, Wetzlar, Germany) with a 300 μm wide blade in the sagittal direction, parallel to the Y axis. The roughness of the cut surface was removed with a polisher (ECOMET3, BUEHLER, Uzwil, Switzerland), and they were polished with waterproof abrasive paper (#400 → #800 → #1200). Specimens for use in microbeam X-ray diffraction system (XRD) were prepared with a thickness of 200 μm.

Three axes were designated in relation to the hyoid bone, with the *X*-axis defined as the long axis of the hyoid body (HB), the *Y*-axis as the direction perpendicular to the long axis of the HB, and the *Z*-axis as the anterior–posterior direction.

### 2.3. Setting of Measurement Sites

**Figure 2:**
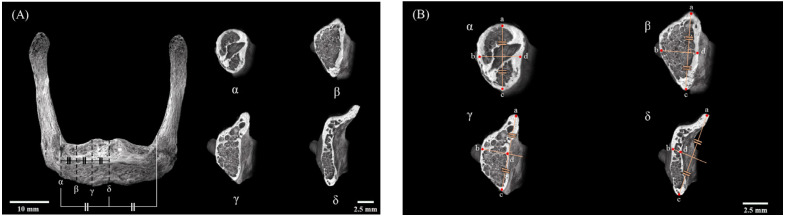
Designation of the region of interest in the human hyoid bone. (**A**) The region of interest was the body of the hyoid between the mesial-most parts of the lesser horns bilaterally. The measurement regions comprised the area of the hyoid body (HB) between the mesial-most part of the lesser horn and the center of the HB divided into four equal parts (α, β, γ, and δ). (**B**) Four points around the circumference of the cortical bone of the HB were used for analysis. Point a; uppermost part of the center of the cortical bone of the HB (superior HB), Point b; anterior-most part of the center of the cortical bone of the HB (anterior HB), Point c; bottommost part of the center of the cortical bone of the HB (inferior HB), Point d; posterior -most part of the center of the cortical bone of the HB (posterior HB) The equals sign (=) indicates lines of equal length.

The region of interest was the body of the hyoid bone. The measurement regions comprised the area between the mesial-most parts of each lesser horn and the center of the HB divided into four equal parts. These quarters were designated as α, β, γ, and δ, respectively (Figure 2A), starting from the mesial-most part of the lesser horn and going toward the center of the HB. In each quarter, the BMD and BAp crystallite orientation were measured at four designated points around the circumference of the cortical bone of the HB. The uppermost part of the center of the cortical bone of the HB was designated as point a (superior HB) and the bottommost part as point c (inferior HB), after which a perpendicular line bisecting the line joining points a and c was drawn, with the ends being designated as point b (anterior HB) and point d (posterior HB) (Figure 2B).

### 2.4. Scanning Electron Microscopy

The cortical bone structure was observed by scanning the cut surfaces of the specimens with a scanning electron microscope (SEM) (SU6600, Hitachi, Tokyo, Japan). The specimen for surface observation was taken from the remaining HB after the sections for evaluation of BAp crystallite orientation were taken. The specimens were polished with waterproof abrasive paper up to #1200 and were treated with Au-Pd sputtering SEM observation. The setting conditions were an acceleration voltage of 25.0 kV and a working distance of 10.9 mm. The measurement points were the sites of interstitial lamellae containing osteons, The measurement points were the sites of interstitial lamellae containing osteons, as BAp crystallite does not exist in the Havers canal [8].

### 2.5. Measurement of Bone Mineral Density

All the specimens were scanned by micro-CT (µCT50, Scanco Medical AG, Wangen-Brüttisellen, Switzerland) with the following scanning conditions: tube voltage 90 kV, tube current 200 μA, matrix size 1024 × 1024, and voxel size 48.4 μm. To observe the internal structure, the images thus obtained were used for three-dimensional (3D) reconstruction using 3D structural analysis software ver. 10.01 (TRI/3D-BON, Ratoc system Engineering Corporation, Tokyo, Japan). For quantitative analysis of BMD (mgHA/cm^3^) of the HB cortical bone at each measurement point, a set of BMD phantoms with X-ray absorption coefficients equivalent to BMDs of 800, 700, 600, 500, 400, 300, and 200 mgHA/cm^3^ (Ratoc System Engineering Corporation, Tokyo, Japan) were scanned with the same condition. The CT gray value of the samples was converted to BMD using the scan data of the BMD phantoms by 3D analysis software ver. 6.6 (Scanco Medical AG, Wangen-Brüttisellen, Switzerland).

### 2.6. Biological Apatite (BAp) Crystallite Orientation

Qualitative analysis of BAp crystallite orientation was performed using an optical-system curved imaging plate (IP) XRD unit (D/MAX RAPIDII-CMF, Rigaku Corporation, Tokyo, Japan). Measurements were made by two methods, one using a transmission-based optical system and the other a reflection-based optical system, with Cu-Kα radiation used as the radiation source in both cases. The device was set to a tube voltage of 40 kV and a tube current of 30 mA. The incident beam was set to a microcircle with a of diameter 100 μm. The diffraction X-ray beam was detected with a curved IP. The measurements for each specimen were used to calculate the X-ray diffraction intensity ratios in the directions of the *X*-, *Y*-, and *Z*-axis for quantitative analysis. The measurement conditions were based on Miyabe et al.’s method for the transmission-based optical system and Nakano et al.’s method for the reflection-based optical system [8,9]. Diffraction intensity along the *X*-axis was measured with the reflection-based optical system, and those along the *Y*- and *Z*-axis were measured with the transmission-based optical system. The incident X-ray beam took account of the Bragg angles of the (002) and (310) peaks. The surface of the specimen was irradiated with the X-ray beam taking into account the Bragg angles θ = 12.95– and 19.9º of the (310) peak. The measurement time at each measurement point was 120 s. The XRD intensity ratio of the two diffraction peaks of the (002) and (310) planes was calculated from the acquired data using two-dimensional data processing software ver. 2.1.6 (2DP, Rigaku Corporation, Tokyo, Japan). This has previously been reported to be a suitable index for evaluating apatite orientation [8,13].

Measurements were conducted thrice at each measurement point, and the mean value was calculated. The XRD intensity ratio of non-oriented apatite powder was 1.53 with the transmission-based system and 1.06 with the reflection-based system.

### 2.7. Statistical Analysis

Two-way analysis of variance (ANOVA) of the mean values for each measurement point was conducted with the measurement region and measurement point as factors, and Tukey’s multiple comparison test was performed. Statistical analysis was carried out using statistical software ver. 4.05 (BellCurve for Excel, Social Survey Research Information, Tokyo, Japan), with *p* < 0.05 regarded as statistically significant.

## 3. Results

### 3.1. Observation of Cortical Bone Surface

**Figure 3:**
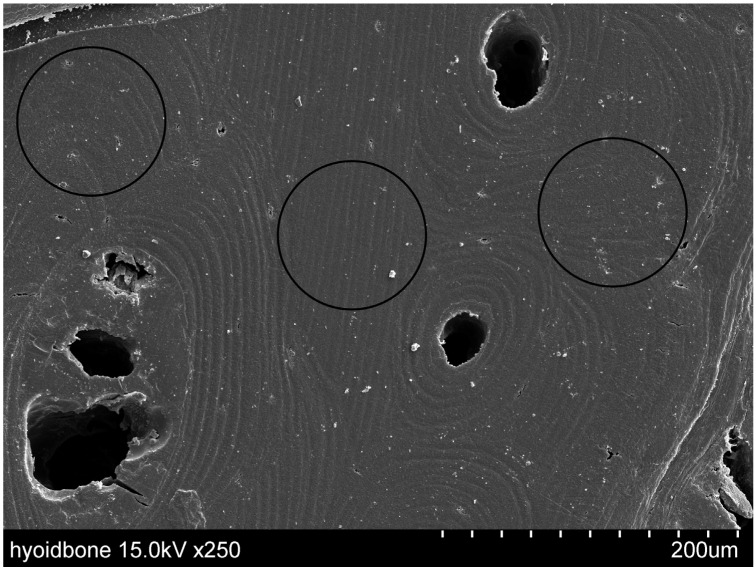
Evaluation of scanning electron microscope (SEM) images.

Figure 3 shows an SEM image of a cross-section of the cortical bone of the HB. Healthy osteons and surrounding interstitial lamellae were evident. This confirmed that the interstitial lamellae could be adequately targeted by an incident beam with a 100 μm diameter.

This SEM image shows the cortical bone of the hyoid body (HB). The black circles represent 100 μm, which is the size of the collimator beam. Osteons and surrounding interstitial lamellae can be seen.

### 3.2. Histological Observation

**Figure 4:**
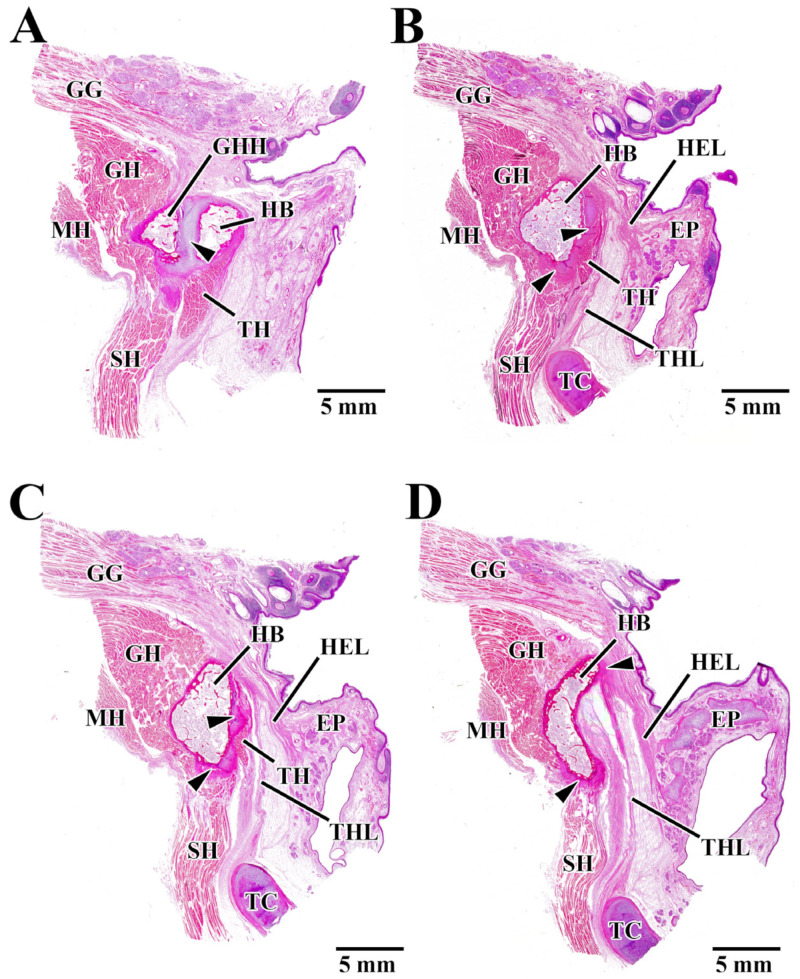
Hematoxylin-eosin-stained sagittal sections from each measurement region (α, β, γ, δ).

We observed muscles and ligaments attached to the HB in the region of interest (Figure 4). In the α region, a septum consisting of cartilaginous tissue was evident between the HB and the greater horn of the hyoid (GHH) (Figure 4A arrowhead). The GH was attached to the anterior and superior parts of the HB, the SH to the inferior part, and the TH to the posterior part (Figure 4A). In the β region, cartilaginous tissue was evident in the inferior and posterior parts of the HB (Figure 4B arrowhead). GH was attached to the anterior part of the HB, SH to the inferior part, and the hyoepiglottic ligament (HEL) and thyrohyoid ligament (THL) to the posterior part (Figure 4B). In the γ region, cartilaginous tissue was evident in the inferior and posterior parts of the HB (Figure 4C arrowhead). The muscle and ligament attachments to the bone were the same as those in the β region (Figure 4C). In the δ region, cartilaginous tissue was evident in the superior and inferior parts of the HB (Figure 4D arrowhead). SH was attached to the inferior part of the HB. TH could not be seen as a muscle attachment to the bone. The attachments of the HEL and THL were localized to the superior and posterior parts where cartilaginous tissue is present (Figure 4D).

A comprehensive evaluation of Figure 4 shows that GH was always attached to the anterior part of the HB, and there was no cartilage intervening between GH and HB. Also, SH was attached to the inferior part of the HB, and cartilage was always present at the SH attachment. TH was attached to the posterior part of the HB. TH attachment contained cartilaginous tissue on the outside of the HB, although the attachment tended to disappear further medially. HEL and THL were attached to the superior part and the superior-posterior part of the HB in the medial panel. A thin layer of cartilage was often present at the attachments of these ligaments. The MH was continuous with the SH and did not attach to the HB. The fact that neither the digastric nor the stylohyoid, the other two suprahyoid muscles, was seen at any of the measurement regions indicated that they were both attached outside the region of interest.

Figure 4A–D show regions α, β, γ, and δ in the hyoid body (HB) region of interest, respectively. Panel A is the most lateral side and panel D is the most median side. In all regions of interest (α, β, γ, δ), the hyoid bone also had muscles or ligaments attached to it. HB; hyoid body, GHH; greater horn of the hyoid, GH; geniohyoid muscle, SH; geniohyoid muscle, TH; thyrohyoid muscle, MH; mylohyoid muscle, GG; genioglossus muscle, HEL; hyoepiglottic ligament, THL; thyrohyoid ligament, TC; thyroid cartilage, EP; epiglottic.

**Figure 5:**
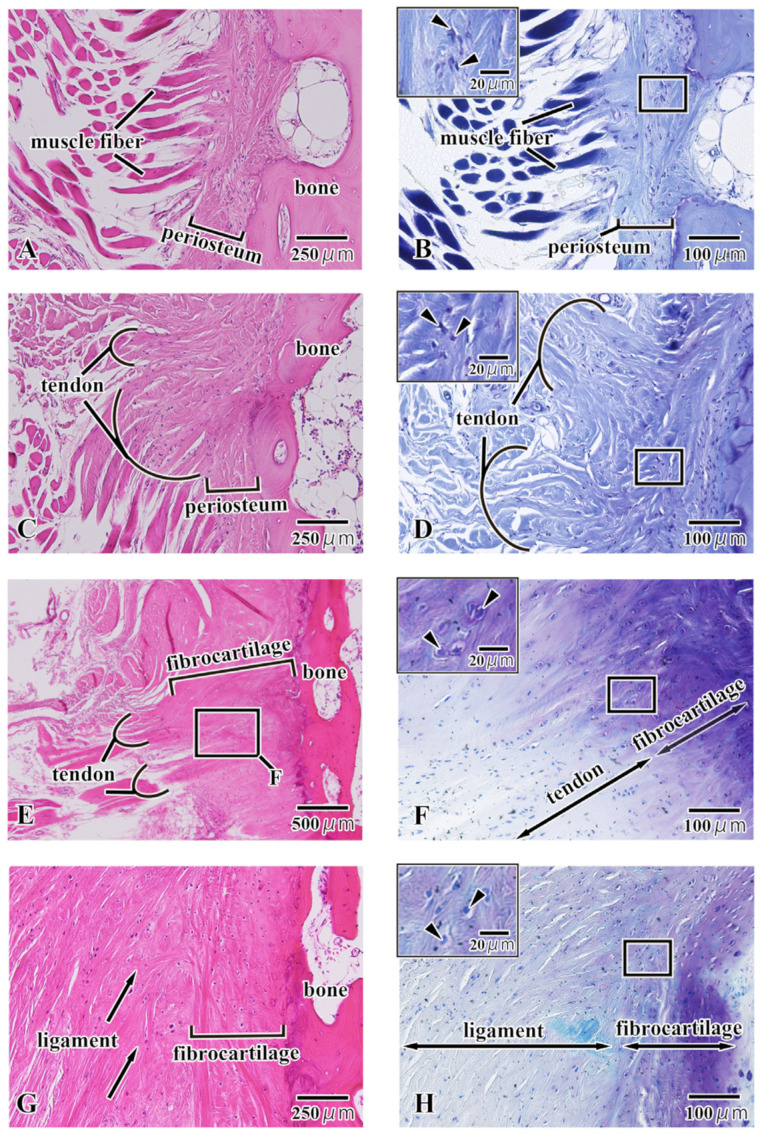
Hematoxylin-eosin-stained and toluidine blue-stained sections showing the morphology of the entheses around the hyoid body (HB).

We observed multiple enthesis patterns within the region of interest (Figure 5). The first pattern was the direct attachment of the muscle fibers to the periosteum. The muscle fibers were not aggregated but were inserted individually into the periosteum (Figure 5A). In the enlarged image of enthesis, there were no metachromatic acid mucopolysaccharides (cartilage substrate: purple) evident within the periosteum (Figure 5B), and the cells within the periosteum were spindle-shaped (Figure 5B small window). The second pattern was the attachment by a tendon via the periosteum. Multiple (fewer than ten) muscle fibers have aggregated to form a tendon, and this tendon was inserted into the periosteum (Figure 5C). In the enlarged image of enthesis, there were no metachromatic acid mucopolysaccharides (cartilage substrate: purple) evident within the periosteum (Figure 5C), and the cells within the periosteum are spindle-shaped (Figure 5D small window). The third pattern was the attachments in which a tendon was transformed into fibrocartilage connecting with the bone. Multiple (several tens of) muscle fibers have aggregated to form a tendon, and the tendon was transformed into fibrocartilage connecting with the bone (Figure 5E). In the enlarged image of enthesis, there were metachromatic acid mucopolysaccharides (cartilage substrate: purple) evident within the fibrocartilage layer (purple) (Figure 5F), and large chondrocytes were present within the lacunae of the cartilage (Figure 5F small window). The fourth pattern was the attachments in which a ligament was transformed into fibrocartilage connecting with the bone. The ligament fibers were inserted obliquely into the fibrocartilage layer, and the individual fibers continue into the bone as if entwining with it (Figure 5G). In the enlarged image of the enthesis, there were metachromatic acid mucopolysaccharides (cartilage substrate: purple) evident within the fibrocartilage layer (purple), and the metachromatic reaction became stronger closer to the bone (Figure 5H). Large chondrocytes were also present within the lacunae of the cartilage (Figure 5H small window).

Direct attachment of the muscle fibers to the periosteum (Figure 5A,B) was localized to the GH–HB attachment (anterior hyoid body). Attachment of a tendon to the periosteum (Figure 5C,D) was seen in every part of the HB (anterior, posterior, superior, and inferior). Entheses of this type were seen supplementing the other types at every location. Attachments in which a tendon (Figure 5E,F) was transformed into fibrocartilage connecting with the bone were observed at the SH–HB (inferior) and TH–HB (posterior) attachments. Attachments in which a ligament was transformed into fibrocartilage connecting with the bone (Figure 5G,H) were observed at the HEL and THL–HB (superior-posterior) attachments. The ligament typically entered at a slant, forming a thin fibrocartilage layer.

Panel A, C, E, and G are H-E-stained images; Panel B, D, F, and H are magnified images of Panel A, C, E, and G, stained with toluidine blue. Direct attachment of the muscle fibers to the periosteum (Panel A and B), attachment by a tendon via the periosteum (Panel C and D), attachments in which a tendon was transformed into fibrocartilage connecting with the bone (Panel E and F), and attachments in which a ligament was transformed into fibrocartilage connecting with the bone (Panel G and H) are shown.

### 3.3. Bone Mineral Density

**Figure 6:**
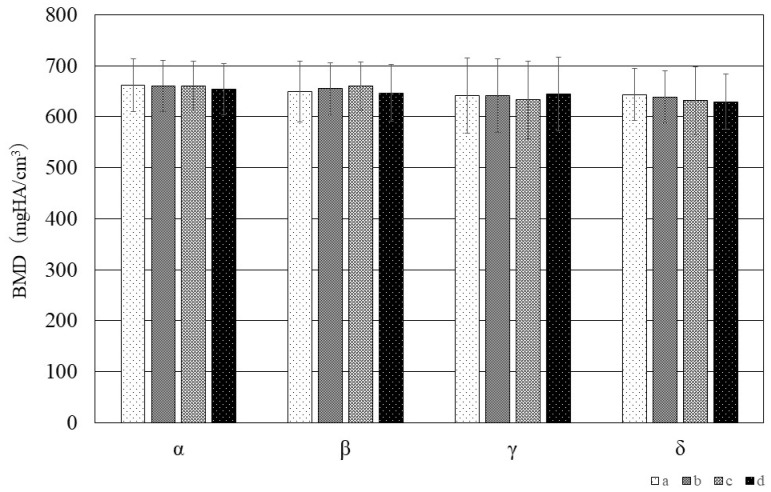
Evaluation of BMD at each measurement point in each measurement region of the hyoid.

Figure 6 shows the results of the quantitative evaluation of BMD at each of the measurement points (a, b, c, d) in each measurement region (α, β, γ, δ). The results of two-way ANOVA revealed no significant difference between any of the measurement regions nor between any of the measurement points.

### 3.4. Bap Crystallite Orientation

**Figure 7:**
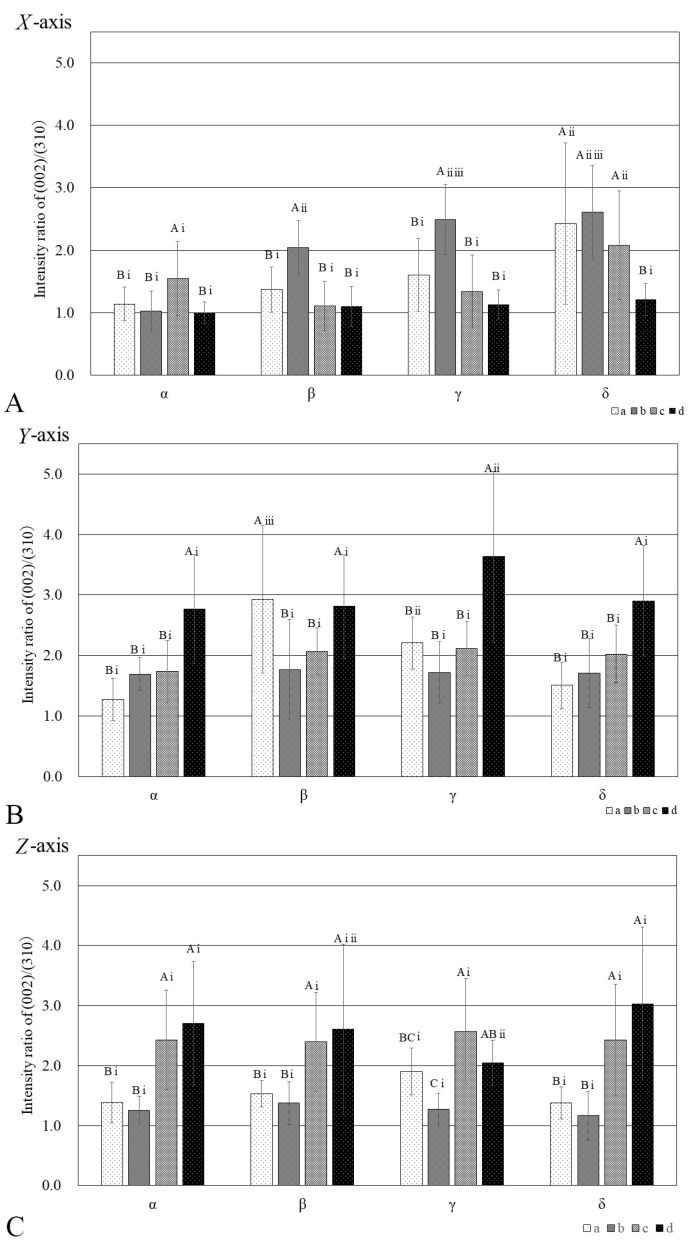
Diffraction intensity ratios along the *X*-, *Y*-, and *Z*-axis, indicating the preferential orientation of BAp at each measurement point in each measurement region of the hyoid.

Figure 7 shows the diffraction intensity ratios along the *X*-, *Y*-, and *Z*-axis, which indicate the preferential orientation of BAp crystallites at each measurement point in each measurement region. The results of two-way ANOVA revealed that a high preferential orientation was observed in the *X*-axis direction at point b in the anterior part of the hyoid body in the β, γ, and δ regions. Compared with the other measurement regions, a high preferential orientation was seen in the δ region at point a in the superior part of the hyoid body and point c in the inferior part of the hyoid body (Figure 7A). In the *Y*-axis direction, a tendency for a high preferential orientation was seen at point d in the posterior part of the hyoid body at all measurement regions (Figure 7B). The highest preferential orientation was evident at point a in the β region. In the *Z*-axis direction, there was a tendency for a high preferential orientation at points c and d at all the measurement regions (Figure 7C) (*p* < 0.05).

The same letter of the alphabet indicates the absence of a significant difference between measurement points, and the same lower-case Roman numeral indicates the absence of a significant difference between measurement regions.

## 4. Discussion

The hyoid bone is located between the mandible and thyroid cartilage and is attached by muscles and ligaments to the base of the skull, the mandible, the tongue, and the larynx. As a result, it is known to be involved in vocalization, respiration, mastication, and swallowing. The hyoid is, therefore, believed to be subjected to forces in a variety of different directions as a result of these functional movements. In particular, the entheses, which represent the attachments where muscles, tendons, and ligaments are inserted into bone, transmit the load imposed by movement to the bone and are thus believed to be the sites of stress concentration [14]. To alleviate this stress concentration and enable efficient load transfer, the tissue layers comprising the entheses vary substantially in composition, structure, and mechanical properties and exhibit complex hierarchical structures [15]. Entheses are generally divided into three types: periosteal insertions, fibrocartilaginous entheses, and fibrous entheses with no intervening fibrocartilaginous layers [15,16,17,18,19].

### 4.1. Local Structure of the Hyoid Body from Histological Observations

The histological observations made in this study revealed periosteal insertions and fibrocartilaginous entheses in the HB. Two types of periosteal insertions in the HB were observed: one with insertion of muscle fibers alone directly into the periosteum and insertions in which multiple muscle fibers fused to form a tendon that was inserted into the periosteum being seen. Direct attachment of the muscle fibers to the periosteum was localized to the attachment from the geniohyoid. Fibrocartilaginous entheses were seen with the SH and TH muscles. This suggests that the entheses of the suprahyoid and infrahyoid muscles might be different. In the trunk and limbs, fibrocartilaginous entheses are seen at the epiphyses of the long bones and at sites where the angle of insertion of the tendon is large, such as the rotator cuff and Achilles tendon, at which the chondrocytes reportedly act as a “stretching brake” that prevents the concentration of stress at the interface with bone [15,20]. The infrahyoid muscles are mainly responsible for pulling the hyoid downwards. They are also reportedly involved in flexing the neck [21]. It has further been suggested that the SH might play an important role in the dilation of the upper airway [22]. Leelamanit et al. have suggested that the TH might play an important role in laryngeal elevation [23]. This muscle is also said to transmit force from the suprahyoid muscles to the cricothyroid complex to open the upper esophageal sphincter [24]. This suggests that, of the infrahyoid muscles, the entheses of the SH and TH muscles in the HB might be sites where the stress generated by these muscle functions is concentrated. Fibrocartilaginous entheses were also observed in the hyoepiglottic and TH ligaments. The hyoepiglottic ligament, which connects the hyoid to the epiglottis, and the TH ligament, which connects the hyoid to the thyroid cartilage, reportedly play important roles in the movement of the epiglottis during the swallowing reflex [25,26,27]. The hyoepiglottic ligament, in particular, is extended during epiglottic movements and is responsible for the movements of bending the epiglottis backward so that it becomes horizontal, and it pulls the tip of the epiglottis forward together with the hyoid, meaning that it plays a major role during the act of swallowing. This suggests that the entheses of these ligaments might thus be sites where the stress generated by swallowing reflex movements is concentrated. However, Vandaele et al. reported that attachment of the hyoepiglottic ligament to the hyoid covers a wide area and that it can be broadly divided into the median hyoepiglottic ligament, which is attached to the hyoid body, and the lateral hyoepiglottic ligament, which is attached to the greater horn [28]. They also reported that the median and lateral hyoepiglottic ligaments have different functions during epiglottic movements and that similar investigations of the greater horn of the hyoid are, therefore, required.

### 4.2. BMD of the Hyoid Body

To our knowledge, although one previous study of the BMD of the hyoid bone has compared measurements in the HB and the greater horn [29], there has been no comparative investigation of localized BMD values in the HB. Our results indicated that there was no significant difference in BMD between any of the measurement regions or measurement points. We previously evaluated the BMD of cortical bone directly below the entheses of the human coronoid process of the mandible and found that there was no significant difference between the BMD of the cortical bone below fibrocartilaginous entheses and fibrous entheses [13]. Previous studies in laboratory animals have also shown that BMD is unaffected by the presence or absence of functional pressure from the muscles [30,31]. This suggests that functional pressure from muscles and ligaments also has a negligible effect on BMD in the human hyoid.

### 4.3. BAp Crystallite Orientation in the Hyoid Body

In our previous study, we reported that BAp crystallite orientation varied depending on the direction of the muscle-tendon attachment [12]. Our results in this study also confirmed that the BAp crystallite preferential orientation varied depending on the measurement region and measurement point. In particular, preferential orientations along the *Y*-axis and *Z*-axis were evident at points c and d (inferior and posterior hyoid body), corresponding to the attachments of the SH and TH muscles and the hyoepiglottic and thyrohyoid ligaments, and these orientations resembled the directions of the courses of the muscles, tendons, and ligaments. The *X*-axis in this study was defined as the long axis of the hyoid bone, and the preferential orientation tended to be more pronounced closer to the center of the hyoid body. At the measurement points, a preferential orientation in the *X*-axis direction was observed in the superior and anterior hyoid body, to which the geniohyoid muscle is attached, as compared to other measurement points. Previous reports have found that long bones such as the femur and ulna reportedly exhibit a uniaxial preferential orientation in the direction of the long axis of the bone [7,8]. Furuya et al. measured BAp crystallite orientation in the cortical bone of human dentulous mandibles and reported that mandibular cortical bone in the vicinity of teeth subject to occlusal pressure exhibited a preferential orientation in the direction of this occlusal pressure, whereas a preferential orientation toward the long axis of the bone was evident at the base of the mandible [10]. In their discussion, they noted that the base of the mandible is horseshoe-shaped and has a structure similar to that of a long bone and that it exhibits a similar preferential orientation to that of long bones in areas not subject to occlusal pressure. Like the mandible, the HB also is horseshoe-shaped and has a structure similar to that of long bones, and as the geniohyoid attachment site has a direct insertion of muscle fibers into the periosteum, the concentration of stress at this site might be comparatively lower than it is at sites with fibrocartilaginous connections, meaning that a uniaxial preferential orientation in the direction of the long axis of the bone was observed.

The above results suggest that the microstructural characteristics of the tendon–bone and ligament–bone attachment sites in the HB might be locally optimized for fibrocartilaginous connections, in order to bear the functional pressure imposed by the infrahyoid muscles and ligaments, that sites with fibrocartilaginous connections have high bone quality in a different direction than the long axis of the bone.

The limitations of this study include the relatively high mean age of the eight Japanese adult cadavers used (82.4 ± 12.1 years), the fact that the number of cadavers was not standardized among the sexes, with five males and three females, and the small sample size. Therefore, we believe it is necessary to increase the number of samples, classify hyoid bones by age and sex, and conduct similar searches for comparison. According to Shimizu et al., the morphology of the hyoid differs depending on age and sex [5]. They also reported that the degree of fusion between the HB and the greater horn increased with age, and the form of the entheses of the hyoid bone and bone quality of the HB might also differ with age and sex. There is also reportedly great variation in the size and shape of the hyoid bone [1], and comparisons of the local structural characteristics of different morphologies might therefore be required.

## 5. Conclusions

Periosteal insertions and fibrocartilaginous entheses are evident at the muscle, tendon, and ligament attachment sites in the human HB. A stronger preferential orientation of BAp crystallites was evident at the sites of infrahyoid muscles and ligaments forming fibrocartilaginous connections than at other sites, and the direction of this orientation was generally consistent with the courses of these tendons and ligaments. This suggests that the functional pressure they impose might have a major effect not only on the morphological characteristics of the entheses but also on bone quality.

## Data Availability

All the data used and analyzed for the current study are contained in the article.
